# Crizotinib in patients with tumors harboring ALK or ROS1 rearrangements in the NCI-MATCH trial

**DOI:** 10.1038/s41698-022-00256-w

**Published:** 2022-03-01

**Authors:** A. S. Mansfield, Z. Wei, R. Mehra, A. T. Shaw, C. H. Lieu, P. M. Forde, A. E. Drilon, E. P. Mitchell, J. J. Wright, N. Takebe, E. Sharon, D. Hovelson, S. Tomlins, J. Zeng, K. Poorman, N. Malik, R. J. Gray, S. Li, L. M. McShane, L. V. Rubinstein, D. Patton, P. M. Williams, S. R. Hamilton, B. A. Conley, C. L. Arteaga, L. N. Harris, P. J. O’Dwyer, A. P. Chen, K. T. Flaherty

**Affiliations:** 1 Division of Medical Oncology, Mayo Clinic, Rochester, MN USA; 2 ECOG-ACRIN Biostatistics Center, Dana-Farber Cancer Institute, Boston, MA USA; 3 Greenebaum Comprehensive Cancer Center, University of Maryland, Baltimore, MD USA; 4 Massachusetts General Hospital, Boston, MA USA; 5 University of Colorado Cancer Center, Aurora, CO USA; 6 Sidney Kimmel Comprehensive Cancer Center at Johns Hopkins, Baltimore, MD USA; 7 Memorial Sloan Kettering Cancer Center and Weill Cornell Medical College, New York, NY USA; 8 Thomas Jefferson University Hospital, Philadelphia, PA USA; 9 Investigational Drug Branch, Division of Cancer Treatment and Diagnosis, National Cancer Institute, Bethesda, MD USA; 10 Cancer Therapy Evaluation Program, Division of Cancer Treatment and Diagnosis, National Cancer Institute, Bethesda, MD USA; 11 Strata Oncology, Inc, Ann Arbor, MI USA; 12 Caris Life Sciences, Irving, TX USA; 13 Tempus, Chicago, IL USA; 14 Biometric Research Program, Division of Cancer Treatment and Diagnosis, National Cancer Institute, Bethesda, MD USA; 15 Center for Biomedical Informatics & Information Technology, National Cancer Institute, Bethesda, MD USA; 16 Frederick National Laboratory for Cancer Research, Frederick, MD USA; 17 City of Hope, Duarte, CA USA; 18 Cancer Diagnosis Program, Division of Cancer Treatment and Diagnosis, National Cancer Institute, Bethesda, MD USA; 19 Simmons Cancer Center, University of Texas Southwestern, Dallas, TX USA; 20 University of Pennsylvania, Philadelphia, PA USA; 21 Division of Cancer Treatment and Diagnosis, National Cancer Institute, Bethesda, MD USA

**Keywords:** Cancer, Cancer

## Abstract

The NCI-MATCH was designed to characterize the efficacy of targeted therapies in histology-agnostic driver mutation-positive malignancies. Sub-protocols F and G were developed to evaluate the role of crizotinib in rare tumors that harbored either *ALK* or *ROS1* rearrangements. Patients with malignancies that progressed following at least one prior systemic therapy were accrued to the NCI-MATCH for molecular profiling, and those with actionable *ALK* or *ROS1* rearrangements were offered participation in sub-protocols F or G, respectively. There were five patients who enrolled on Arm F (ALK) and four patients on Arm G (ROS1). Few grade 3 or 4 toxicities were noted, including liver test abnormalities, and acute kidney injury. For sub-protocol F (ALK), the response rate was 50% (90% CI 9.8–90.2%) with one complete response among the 4 eligible patients. The median PFS was 3.8 months, and median OS was 4.3 months. For sub-protocol G (ROS1) the response rate was 25% (90% CI 1.3–75.1%). The median PFS was 4.3 months, and median OS 6.2 months. Data from 3 commercial vendors showed that the prevalence of *ALK* and *ROS1* rearrangements in histologies other than non-small cell lung cancer and lymphoma was rare (0.1% and 0.4% respectively). We observed responses to crizotinib which met the primary endpoint for *ALK* fusions, albeit in a small number of patients. Despite the limited accrual, some of the patients with these oncogenic fusions can respond to crizotinib which may have a therapeutic role in this setting.

## Introduction

The NCI MATCH trial is an expansive National Clinical Trials Network/NCI Community Oncology Research Program effort, developed and implemented with the goal of providing a large-scale platform for the study of targeted agents in molecularly defined malignancies. With the availability of molecular testing and drugs designed to target actionable mutations, this effort has been one of the largest to provide access to treatment based on driver mutation status, rather than histology or primary site of disease. The chimeric proteins that result from gene fusions involving *ALK* or *ROS1* are both therapeutic targets of the tyrosine kinase inhibitor crizotinib. The kinase domains of ALK and ROS1 share significant amino homology within the ATP-binding sites, resulting in high-affinity binding of crizotinib in cell-based assays. The role that ALK plays in normal physiology is poorly understood. In the wild-type state, activation of the ALK protein is thought to be potentially mediated by ligand-induced dimerization. Oncogenic activation of ALK arises from a rearranged protein in which the intact tyrosine kinase domain of ALK is fused to a variety of upstream partners resulting in constitutive activation of the ALK tyrosine kinase. This, in turn, results in activation of downstream signaling involving the MAPK, PI3K, and JAK/STAT pathways, thereby promoting increased cell growth and proliferation^1^.

In non-small cell lung cancer (NSCLC) *EML4-ALK* is one of the more common fusion events, but there are other reported variants of ALK fusion proteins that do not involve the *EML4* gene such as *KIF5B-ALK*, *STRN-ALK*, *HIP1-ALK*, *VCL-ALK*, *NPM-ALK,* and *TGF-ALK*^2–5^. *ALK* fusions have been identified as drivers of oncogenesis in a variety of other malignancies^6^. *ALK*-positive anaplastic large cell lymphomas (ALCLs) represent a distinct subset of lymphomas that are associated with better outcomes in comparison to *ALK*-negative ALCLs. Up to 50% of *ALK*-positive ALCLs harbor *ALK* fusions, the most common of which is the t(2;5)(p23;q35) translocation, resulting in the formation of *NPM-ALK*. In solid tumors, *ALK* rearrangements including *TPM4-ALK* and *TPM3-ALK* have been identified in up to 50-75% of inflammatory myofibroblastic tumors (IMT). These fusions are transforming in various cell lines and animal models. Recently, *ALK* rearrangements have been found in spitzoid neoplasms, a group of melanocytic tumors including Spitz nevi, spitzoid melanomas, and atypical Spitz tumors^7^.

*ROS1* encodes a transmembrane protein with an extracellular domain that is partially analogous to fibronectin^8^. Although the function of wild-type *ROS1* is poorly understood, it is speculated that ROS1 might translate adhesion events to intracellular signaling because of the structural similarities to cell adhesion molecules. In NSCLC, the most common *ROS1* fusion partner is *CD74*. Less common partners include *SDC4*, *EZR*, *SLC34A2*, *TPM3*, *LIMA1*, and *MSN*. The *ROS1* proto-oncogene has been identified to be translocated in NSCLC with a frequency of 1.7% (18/107) to 2.6% (17/656)^9,10^. While rare, *ROS1* rearrangements have also been reported in gastric cancers, glioblastomas, cholangiocarcinomas, ovarian cancers, colorectal cancers, inflammatory myofibroblastic tumors, angiosarcomas, and epithelial hemangioendotheliomas^8,11,12^.

Crizotinib is a first-in-class ATP-competitive small-molecule inhibitor of ALK, ROS1, and Met/hepatocyte growth factor receptor (HGFR). Crizotinib demonstrated concentration dependent inhibition of ALK kinase activity in biochemical and cell-based assays. In addition, crizotinib demonstrated growth inhibition and increased apoptosis of tumor cell lines with *ALK* fusion variants (*EML4-ALK* or *NPM-ALK*). Mice xenografts with *ALK* fusion variants also responded to crizotinib treatment in a dose dependent fashion. Based on its significant activity, crizotinib received FDA approval for *ALK*-positive NSCLC in 2011, for *ROS1*-positive NSCLC in 2016, and *ALK*-positive ALCL in 2021. In prior studies, common toxicities related to crizotinib included reversible visual disturbances, gastrointestinal side effects, fatigue, transaminitis and edema.

The NCI-MATCH subprotocols F and G were designed to study the activity of crizotinib in tumors other than NSCLC or ALCL with *ALK* and *ROS1* rearrangements, respectively. Given the common therapeutic option in these subprotocols, the results of both studies were combined for this report.

## Results

### Patient characteristics

A total of 5 patients enrolled on sub-protocol F (ALK), with the first patient enrolled on November 24, 2015, and the last on April 24, 2019. One patient was ineligible since treatment was started before a 28-day washout following prior treatment, resulting in four analyzable patients (Supplemental Fig. 1). A total of 4 patients enrolled on sub-protocol G (ROS1), with the first patient enrolled on July 7, 2016, and the last on September 13, 2018 (Supplemental Fig. 2). Due to poor accrual, both sub-protocols were closed. Among both sub-protocols, most patients had gastrointestinal malignancies (Table 1). All but one patient had received two or more prior lines of therapy. Previously reported *EML4* and *GOPC* fusion partners were most frequently identified for *ALK* and *ROS1*, respectively. A median of four cycles of crizotinib were administered in both sub-protocols.

**Table 1:** Patient Characteristics.

	*ALK* fusion (*n* = 4)	*ROS1* fusion (*n* = 4)
Female	3 (75%)	2 (50%)
Age: median (range)	59 (52–69) years	54 (47–76) years
Race: White	4 (100%)	4 (100%)
Performance Status 0	3 (75%)	2 (50%)
1	1 (25%)	2 (50%)
Number of Prior Therapies: 1	1 (25%)	0 (0%)
2	2 (50%)	1 (25%)
3	0 (0%)	1 (25%)
>3	1 (25%)	2 (50%)
Weight loss previous 6 months: < 5%	4 (100%)	4 (100%)
Histologies		
Cholangiocarcinoma		1
Pancreatic adenocarcinoma		1
Colorectal adenocarcinoma	2	1
Ovarian adenocarcinoma		1
Carcinoma of unknown primary	1	
Leiomyosarcoma	1	
Fusion partners		
*EML4*	3	
*ACTG2*	1	
*STRN*	1	
*GOPC*		4

### Response assessment and survival outcomes

**Fig. 1:**
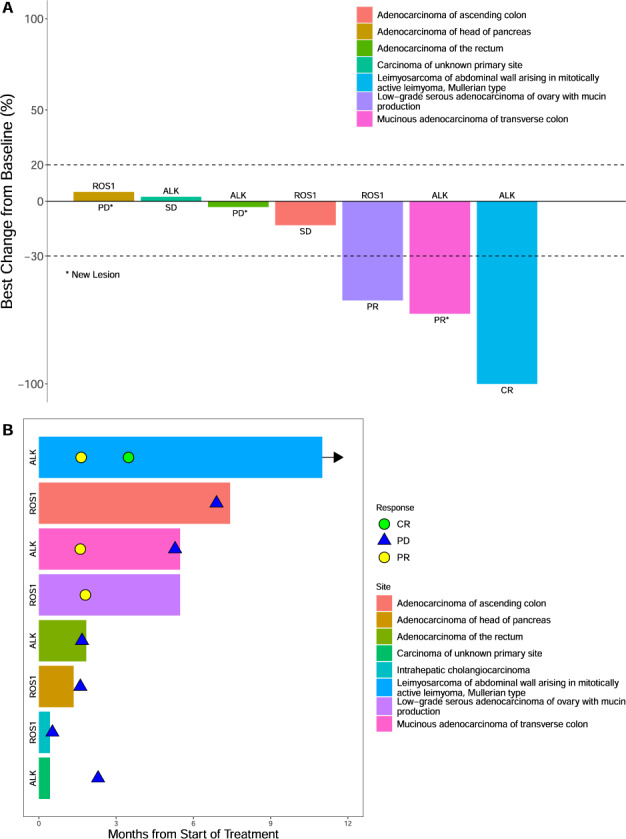
Waterfall and Swimmer plot of responses and their durations. The waterfall plot shows responses for all patients who had response assessment (**A** *n* = 7). One patient on subprotocol G (ROS1) is not included as treatment was discontinued during cycle one for toxicity and response was not evaluable. The Swimmer plot shows the duration of responses for all patients (**B** *n* = 8). *CR* complete responses, *PD* progressive disease, *PR* partial response.

**Fig. 2:**
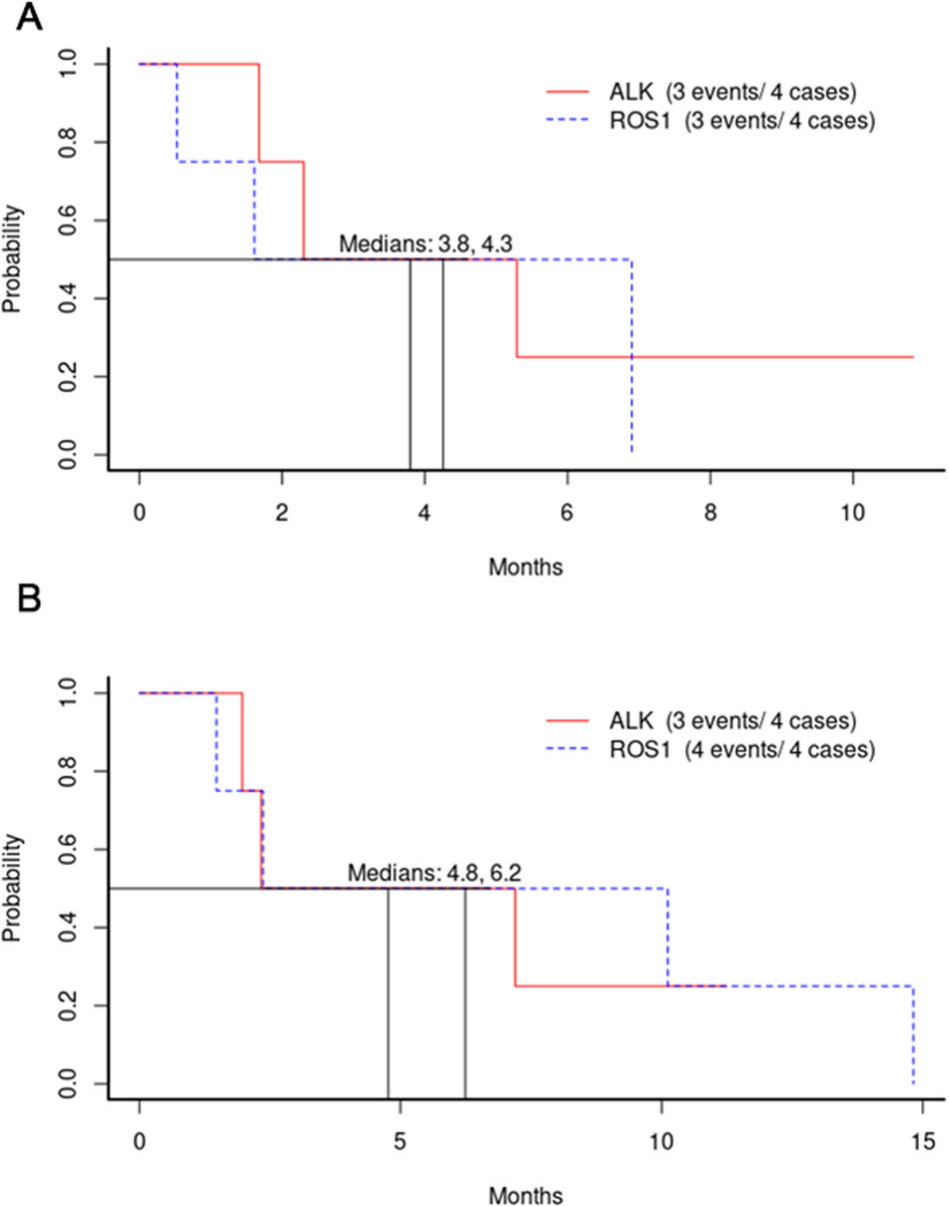
Progression-free and overall survival for both sub-protocols. The PFS **A** and OS **B** are presented for both ALK (EAY131-F) and ROS1 (EAY131-G) sub-protocols.

For sub-protocol F (ALK), the response rate was 50% (90% CI 9.8–90.2%; Fig. 1) with one, ongoing complete response; the median PFS was 3.8 months, and the 6-month PFS rate was 25% (90% CI 6.0%-100%; Fig. 2). Despite the very low accrual, the null hypothesis of response rate not exceeding 5% can be rejected at the 1-sided .014 significance level, meeting the primary endpoint. The median overall survival was 4.8 months (Fig. 2; Supplemental Table 3). For sub-protocol G (ROS1), with a 25% responses rate (90% CI 1.3–75.1%; Fig. 1), the *p*-value for testing the null of 5% was 0.186. The median PFS was 4.3 months, and the 6-month PFS rate was 50% (90% CI 22–100 %; Fig. 2). The median overall survival was 6.2 months (Fig. 2; Supplemental Table 3).

### Adverse events

There were very few grade 3 or 4 toxicities at least possibly related to treatment; these included liver test abnormalities, abdominal pain, and acute kidney injury. Less severe edema, hypoalbuminemia, and liver test abnormalities were also observed (Supplemental Table 4). There were two deaths on sub-protocol F attributed to disease progression or a cause that was not otherwise specified. There was one death on sub-protocol G attributed to disease progression.

### Expanded molecular cohort

**Fig. 3:**
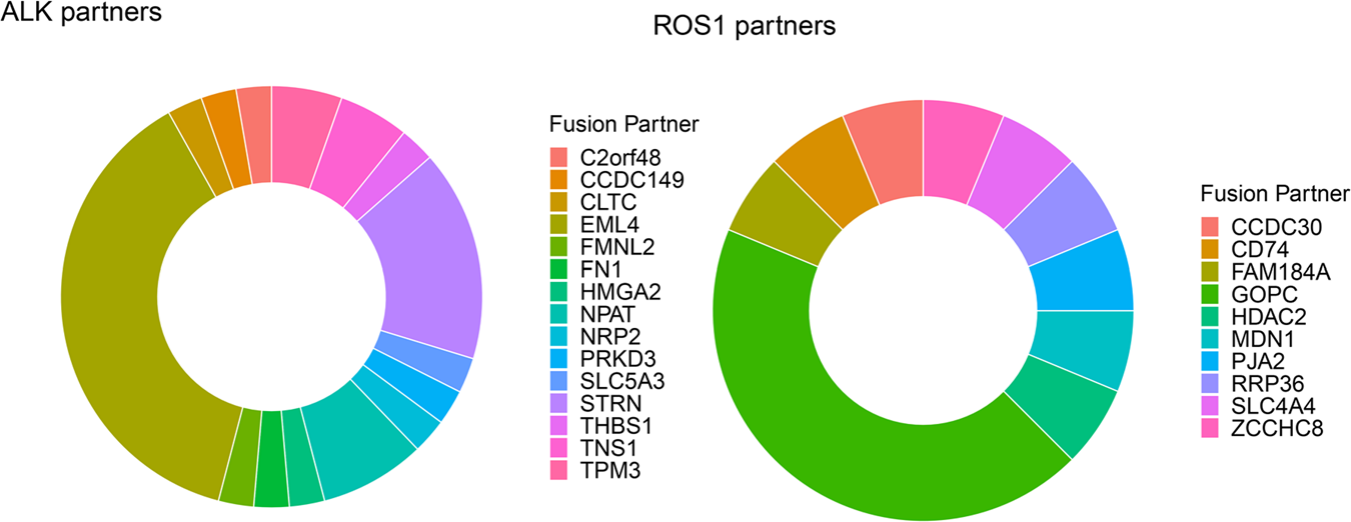
Fusion partners for *ALK* and *ROS1* in the expanded molecular cohort. The most common fusion partners are shown for *ALK* and *ROS1* from the expanded molecular cohort.

In an expanded molecular cohort of subjects compiled by three commercial vendors, the detection of *ALK* or *ROS1* rearrangements outside of NSCLC or lymphoma was rare (Supplemental Table 5). A review of over 30,000 tumors excluding those with NSCLC or lymphoma from two of these commercial vendors identified *ALK* rearrangements in 1/1,000 specimens and *ROS1* rearrangements in 4/10,000 specimens. One external dataset included clinical characteristics of these cases. Whereas there was a similar proportion of females (*n* = 18, 49%) and males (*n* = 19, 51%) with *ALK* rearrangements, there were more females (*n* = 10, 63%) than males (*n* = 6, 37%) with *ROS1* rearrangements. Although there were similar median ages for patients with either rearrangement, there were younger patients with *ALK* rearrangements than *ROS1* rearrangements (*ALK* median 57 years, range 25–83; *ROS1* median 60.5 years, range 41–89). *ALK* rearrangements were most frequently seen in thyroid, colorectal, and soft tissue tumors in this cohort (Supplemental Table 6). *ROS1* rearrangements were most frequently seen in glioblastoma multiforme and breast cancer (Supplemental Table 7). The most frequent *ALK* fusion partners were *EML4* (38%) and *STRN* (16%) and the most frequent *ROS1* fusion partner was *GOPC* (44%) (Fig. 3). Of the evaluable tumors, almost all with *ALK* or *ROS1* fusions had low or intermediate TMB (96%; Supplemental Table 8) and were MSI stable (98%; Supplemental Table 9).

## Discussion

Accrual to sub-protocols F and G was limited, reducing our ability to assess the efficacy of treatment with any precision. However, we can conclude that the true response rate for sub-protocol F exceeds the null hypothesis rate of 5%, at the 1-sided .014 significance level. We observed responses to crizotinib in patients whose tumors harbored *ALK* or *ROS1* rearrangements outside of the FDA-approved indications for this therapy. However, the median overall survivals of 4.8 and 6.2 months suggest that the anti-tumor activity we observed was modest. Many of the reported toxicities were similar to those reported in prior trials with crizotinib. Furthermore, our rates of detection and analysis of external datasets from three commercial vendors suggest that *ALK* and *ROS1* rearrangements are very rare outside of NSCLC.

Globally, there have been additional efforts to target either ALK or ROS1 in rare tumor types. Some of the more successful attempts have been in the pediatric population. For instance, response rates as high as 80–90% with ALK inhibitor therapy have been observed in the treatment of anaplastic large cell lymphomas which harbor *ALK* translocations^13,14^. Inflammatory myofibroblastic tumors are another rare malignancy that frequently harbor *ALK* or *ROS1* translocations^15–17^. In one case, the *ALK* rearrangement resulted from a complex pattern of chromosomal rearrangements called chromoplexy, and that patient had durable benefit from treatment with the ALK inhibitor ceritinib^18^. Finally, molecular events involving *ALK* have also been identified in aggressive thyroid cancers and neuroblastomas^19,20^. While the role of ALK and ROS1 inhibitors is now established as standard of care among *ALK* - or *ROS1* -positive NSCLC in addition to some of the rare tumors listed above, our data suggest that patients with other tumor types that harbor these mutations may also benefit from targeted therapy. The authors are not aware of other plans to develop crizotinib further for these indications.

There are several potential explanations for the modest impact on response rate and survival noted in both sub-protocols. First, this was a pre-treated population with most patients having received at least 2 lines of prior therapy. As a result, it is likely that these patients had more resistant tumors at the time of treatment. Somatic mutations that confer resistance to crizotinib are now well characterized among series of patients with NSCLC and acquired resistance mutations in *ALK* include L1196M, G1202R, and S1206Y^21^. Alternatively, these patients’ tumors may have developed bypass pathway signaling or had concurrent mutations as a result of prior therapy which limited the activity of crizotinib. One patient had a concurrent *BRAF* fusion, which may have been the actual oncogenic driver. Since the approval of crizotinib, more potent ALK and ROS1 inhibitors have received FDA approval such as alectinib^22^, brigatinib^23^, entrectinib^24^, and lorlatinib^25,26^. Enrollment to basket trials with some of these agents may have competed with enrollment to NCI-MATCH. At this time, it is not known if one of these newer agents would yield improved efficacy in these other tumor types with *ALK* or *ROS1* fusions. Finally, it has been suggested that sequencing RNA might be more appropriate than DNA for the detection of fusions given the difficulties of covering all of the introns from which rearrangements can arise^27^. The central NCI-MATCH NGS assay did sequence fusions from RNA input, but the assay was specifically targeted for known fusions. The external laboratories use a variety of platforms but many scan introns in DNA. For that reason, there may have been some cases with novel *ALK* or *ROS1* fusions that were not detected for inclusion in NCI-MATCH.

Our analysis of the expanded molecular cohort identified *ALK* or *ROS1* rearrangements in many types of solid tumors. The frequencies of fusion partners did not necessarily match those reported for non-squamous NSCLC but included many of the known fusion partners. Almost all the tumors in the expanded cohort with *ALK* or *ROS1* rearrangements had low or intermediate TMB and were microsatellite-stable, suggesting that there are few other molecular drivers in these cases.

The NCI-MATCH is ongoing with open sub-protocols. Recently, the experience performing molecular profiling on almost 6,000 patients for NCI-MATCH was reported^28^. Thirty subprotocols were open at some point during this screening period and eleven of them met their accrual goals of 31 eligible patients. None of the subprotocols that targeted an alteration with a prevalence <1.5% met its accrual goal during this centralized screening phase. These results highlight the difficulties of identifying eligible patients with rare molecular events for clinical trial participation despite the widespread activation of NCI-MATCH and significant engagement with community oncologists. It is possible that just-in-time clinical trial activation mechanisms could have expanded the number of potential sites with eligible patients and improved accrual to subprotocols with rare molecular events. There was a higher rate of molecular alterations and co-occurring mutations in NCI-MATCH than seen in TCGA^29^, which may have resulted from patient selection strategies and larger sample size for some tumor types like cholangiocarcinoma in NCI-MATCH. Many of the co-occurring mutations were in tumor-suppressor genes that have been implicated in therapeutic resistance to targeted therapies. Given the genomic complexity of many cancers and the resistance that invariably develops with most single agent targeted therapies, the NCI-ComboMATCH (EAY191)^30^ will test combinations of targeted therapies that are supported by robust in vivo evidence.

In spite of the low incidence of *ALK* and *ROS1* rearrangements among adults with solid tumors other than NSCLC, we feel it is still critical to pursue comprehensive molecular testing for rare tumors and those with limited treatment options. Ongoing evaluation of newer inhibitors is needed in the rare event of an *ALK* or *ROS1* translocation. Given rarity of these events in adult solid tumors, it will require collaboration amongst the larger oncology community to study the efficacy of these agents in this setting.

## Methods

### Study design and eligibility

The NCI-MATCH trial is an ongoing, nationwide clinical trial with integrated multiple independent single-arm sub-protocols, each addressing an actionable molecular alteration. Each sub-protocol aims to evaluate a single agent or combination treatment for which at least a recommended phase 2 dose has been determined.

Patients with histologically documented solid tumors, lymphomas, or myelomas whose disease had progressed following at least one line of standard systemic therapy or for whom no standard therapy exists were registered on the screening step of the NCI-MATCH protocol to undergo molecular profiling analysis on fresh tumor biopsies. The latter profiling was performed in specific Clinical Laboratory Improvement Amendments (CLIA)-accredited laboratories and consisted of next generation sequencing (NGS) with an investigational targeted gene panel, the Oncomine Ampliseq assay^31^. This assay was validated to detect specific targeted gene fusions with 97.67% sensitivity and 99.99% specificity. In the second phase of the trial, 30 academic and commercial laboratories that perform NGS testing were reviewed and evaluated for concordance of test results with the central NCI-MATCH laboratory assay. These laboratories were then asked to refer patients with an actionable mutation for enrollment. Patients whose tumors were found to harbor actionable *ALK* or *ROS1* rearrangements (Supplementary Tables 1-2) were offered participation in sub-protocols F or G, respectively. The protocol allowed for subsequent treatment on other subprotocols if relevant molecular profiles were present and eligibility criteria were met. The study was initiated after approval from the Central Institutional Review board and was conducted in accordance with the Declaration of Helsinki. Written informed consent was obtained from all patients.

Patients with NSCLC were excluded from both sub-protocols, given that the FDA previously approved crizotinib for patients with NSCLC harboring *ALK* or *ROS1* rearrangements. Patients who had already received ALK or ROS1 inhibitors were also excluded. Patients were treated with crizotinib 250 mg twice daily on 28-day cycles. Dose reductions to 200 mg twice daily or 250 mg daily were allowed for treatment-related adverse events. Responses were assessed using revised RECIST (Response Evaluation Criteria in Solid Tumors) version 1.1^32^, Cheson criteria for patients with lymphoma,^33^ and RANO criteria for patients with glioblastoma multiforme^30^.

### Statistical considerations

The primary objective of these NCI-MATCH sub-protocols was to evaluate the proportion of patients who had objective response, defined as complete or partial response, to a targeted study agent. This proportion, termed the objective response rate and expressed as a percentage, was compared against a null benchmark value of 5%. If the observed objective response rates were ≥ 5/31 (16%), it would then be concluded that the agent is promising and worthy of further investigation. Allowing for a 10% ineligibility rate, 35 patients were to be accrued to each sub-protocol, to obtain 31 eligible patients per sub-protocol. With this design, the power was 91.8% to conclude an agent is promising if its true response rate is 25%, and the type 1 error (one-sided) was 1.8% of its true response rate is 5%. Secondary objectives included the proportion of patients who were progression-free at 6 months (PFS6), progression-free survival, and toxicity assessment.

### Expanded molecular cohort

Three NGS vendors provided data on the detection of *ALK* and *ROS1* rearrangements in the tumors of patients excluding NSCLC and lymphoma as part of their commercial testing services in an effort separate from NCI-MATCH. We used these data to see how the frequency of *ALK* and *ROS1* rearrangements in this expanded molecular cohort compared to that detected by NCI-MATCH. Two of these vendors provided the total number of cases tested, and one vendor provided additional data on the clinical characteristics of these tumors, microsatellite instability (MSI) status, and tumor mutation burden (TMB). These cases were not necessarily included in NCI-MATCH. MSI was examined using over 7,000 target microsatellite loci and compared with the reference genome hg19 from the University of California, Santa Cruz (UCSC) Genome Browser database. The status was defined as MSI-high (MSI-H) or MSI-low/microsatellite stable (MSS). The number of microsatellite loci that were altered by somatic insertion or deletion were counted for each sample. Only insertions or deletions that increased or decreased the number of repeats were considered. Genomic variants in the microsatellite loci were detected using the same depth and frequency criteria used for mutation detection. MSI-NGS results were compared with results from over 2,000 matching clinical cases analyzed with traditional PCR-based methods. The threshold to determine MSI by NGS was determined to generate a sensitivity of >95% and specificity of >90%. TMB was measured by counting all nonsynonymous missense mutations found per tumor that had not been described previously as germline alterations [592 genes and 1.4 megabases (MB) sequenced/tumor]. Potential germline mutations were excluded by comparing data against dbSNP 137 full and 1000 Genomes Phase 3. The threshold to define TMB-high (TMB-H) was ≥17 mutations/MB and was established by comparing TMB with MSI by fragment analysis in colorectal cancer cases, based on reports of TMB having high concordance with MSI-H in colorectal cancer. TMB-intermediate was defined as ≥ 7 but <17 mutations/megabase, and TMB-low was defined as ≤6 mutations/megabase. These data were summarized and the donut charts were created with R Studio (version 1.2.5033) and tidyverse packages.

### Reporting summary

Further information on research design is available in the Nature Research Reporting Summary linked to this article.

## Supplementary information

Supplemental Material

Supplemental Table

REPORTING SUMMARY

## Data Availability

The data generated or analysed from these clinical trials are included in this published article. The expanded molecular datasets generated or analysed for the current study are available from the corresponding author on reasonable request. The deidentified sequencing data from Caris Life Sciences are owned by Caris Life Sciences. Qualified researchers can apply for access to these summarized data by contacting Joanne Xiu, PhD and signing a data usage agreement. Strata Oncology will provide de-identified molecular results upon request for Strata-referred samples included in this analysis. More specifically, they will provide all prioritized variants for the ALK/ROS1 + patients, including for the fusions 5′/3′ partners, junctions and read support, as well as cancer type, age range, etc. Requests should be addresses to Dan Hovelson, PhD. Tempus has provided summary statistics, including the number of screened patients for this cohort and the count of the specific positives for post-publication replication and verification purposes (Supplemental Table 10). The protocols are available as supplementary materials.
