# Supplementary material for: Crizotinib in patients with tumors harboring ALK or ROS1 rearrangements in the NCI-MATCH trial
Source: NPJ Precis Oncol. 2022 Mar 1;6:13. doi: 10.1038/s41698-022-00256-w (PMC8888601; doi:10.1038/s41698-022-00256-w)
Supplement: Supplementary file 1 — Supplemental Material [file 41698_2022_256_MOESM1_ESM.pdf]

Supplemental Figure 1: Sub-protocol F (ALK) CONSORT diagram

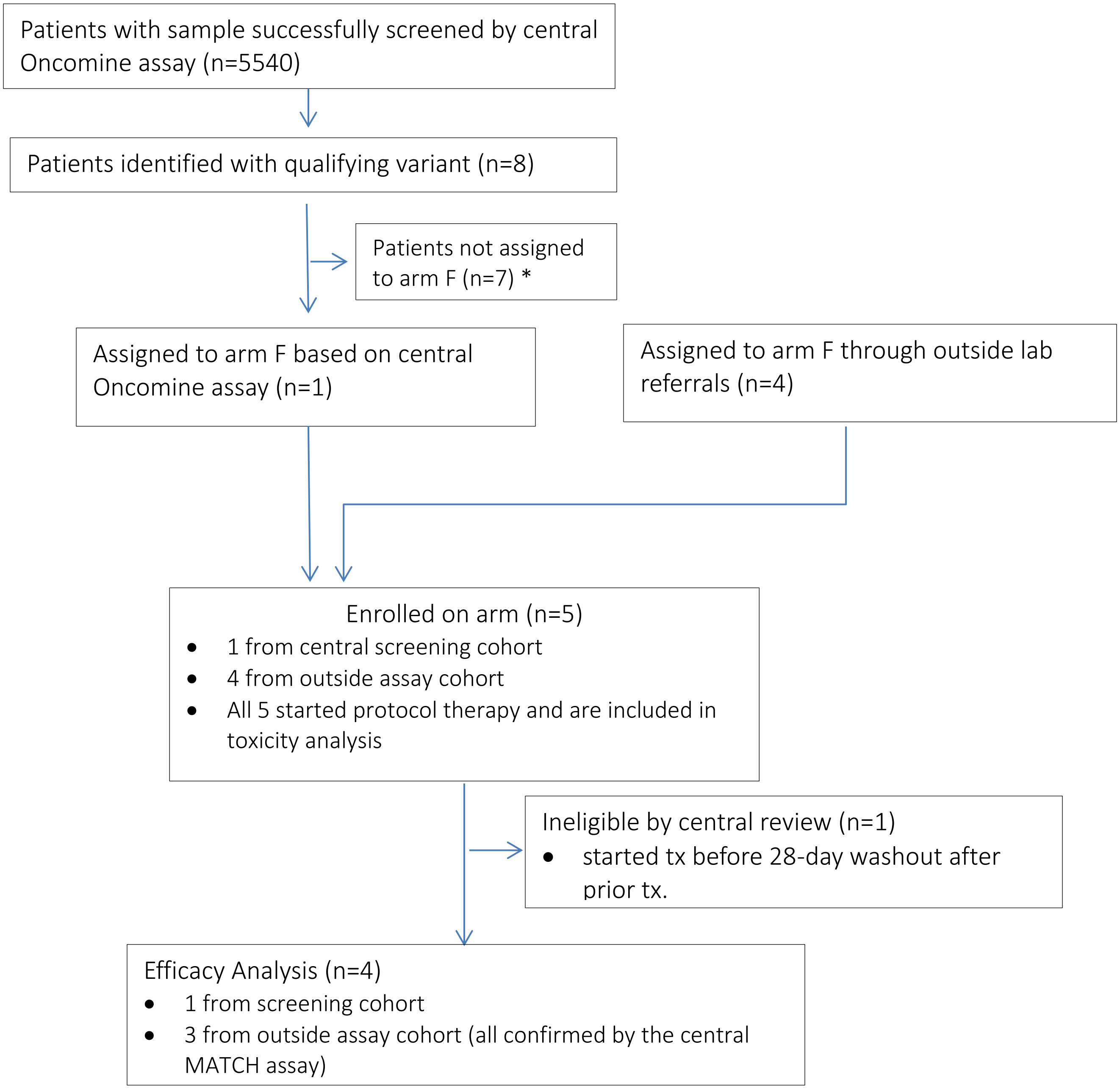

*Reasons not receiving an assignment (all due to ineligible histology): Lung adenocarcinoma (n=7)

Supplemental Figure 2: Sub-protocol G (ROS1) CONSORT diagram

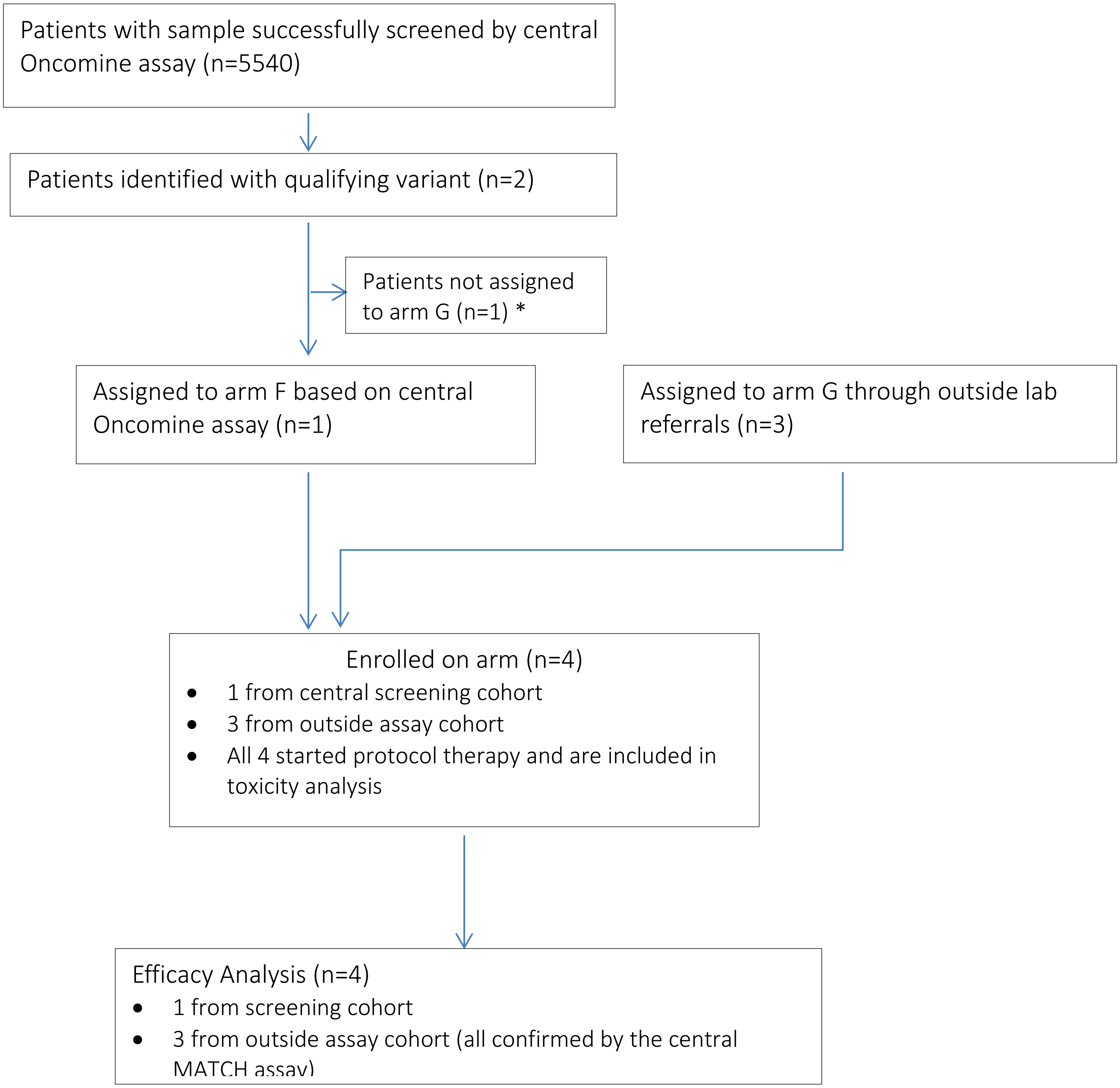

*Reasons not receiving an assignment: Not meeting the requirements of Section 3.1 of the Match Master Protocol: Patients must have ECOG performance status <= 1 (n=1)

Supplemental Table 1: Actionable Mutations for Sub-Protocol EAY131-F

Gene	Variant ID
ALK	ACTG2-ALK.A2A18
ALK	C2orf44-ALK.C4A20.COSF1264
ALK	CLIP4-ALK.C11A23
ALK	EML4-ALK.E13A20.AB462411
ALK	EML4-ALK.E13A20.COSF1062
ALK	EML4-ALK.E13A20.COSF408
ALK	EML4-ALK.E14A20.COSF1064
ALK	EML4-ALK.E14A20.COSF477
ALK	EML4-ALK.E15A20.COSF413
ALK	EML4-ALK.E17A20.COSF1366
ALK	EML4-ALK.E17A20.COSF1367
ALK	EML4-ALK.E18A20.COSF487
ALK	EML4-ALK.E18A20.COSF487.1
ALK	EML4-ALK.E20A20.COSF409
ALK	EML4-ALK.E20A20.COSF730
ALK	EML4-ALK.E2A20.COSF478
ALK	EML4-ALK.E2A20.COSF479
ALK	EML4-ALK.E6A17
ALK	EML4-ALK.E6A18
ALK	EML4-ALK.E6A19.COSF1296
ALK	EML4-ALK.E6aA20.AB374361
ALK	EML4-ALK.E6aA20.COSF411
ALK	EML4-ALK.E6bA20.AB374362
ALK	EML4-ALK.E6bA20.COSF412
ALK	EML4-ALK.E7A20.NGS
ALK	GTF2IRD-ALK.G7A20
ALK	HIP1-ALK.H21A20
ALK	HIP1-ALK.H28A20
ALK	KIF5B-ALK.K15A20.COSF1060
ALK	KIF5B-ALK.K15A20.COSF1381
ALK	KIF5B-ALK.K17A20.COSF1257
ALK	KIF5B-ALK.K24A20.COSF1058
ALK	KLC1-ALK.K9A20.COSF1276
ALK	MEMO1-ALK.M2A7
ALK	NCOA1-ALK.N21A1.NGS
ALK	PRKAR1A-ALK.P2A20.NGS
ALK	PTPN3-ALK.P2A10
ALK	STRN-ALK.S3A20.COSF1430
ALK	TFG-ALK.T4A20.COSF424
ALK	TFG-ALK.T5A20.COSF426

ALK	TFG-ALK.T6A20.COSF428
ALK	TPM1-ALK.T8A20.NGS
ALK	TPM3-ALK.T7A20
ALK	TPM4-ALK.T6A20
ALK	TPR-ALK.T15A20
ALK	VCL-ALK.V16A20.COSF1057

Supplemental Table 2: Actionable Mutations for Sub-Protocol EAY131-G

Gene	Variant ID
ROS1	CCDC6-ROS1.C5R35
ROS1	CD74-ROS1.C4R33.NGS
ROS1	CD74-ROS1.C6R32.COSF1202
ROS1	CD74-ROS1.C6R34.COSF1200
ROS1	CD74-ROS1.C6R35
ROS1	CEP85L-ROS1.C8R36
ROS1	CLIP1-ROS1.C19R36
ROS1	CLTC-ROS1.C31R35
ROS1	ERC1-ROS1.E11R36
ROS1	EZR-ROS1.E10R34.COSF1267
ROS1	EZR-ROS1.E10R35
ROS1	GOPC-ROS1.G4R36.COSF1188
ROS1	GOPC-ROS1.G8R35.COSF1139
ROS1	HLA_A-ROS1.H7R34
ROS1	KDELR2-ROS1.K5R35
ROS1	KDELR2-ROS1.K5Rintron34
ROS1	KIAA1598-ROS1.K11R36
ROS1	LRIG3-ROS1.L16R35.COSF1269
ROS1	MSN-ROS1.M9R34
ROS1	MYO5A-ROS1.M23R35
ROS1	PPFIBP1-ROS1.P9R35
ROS1	PWWP2A-ROS1.P1R36
ROS1	SDC4-ROS1.S2R32.COSF1265
ROS1	SDC4-ROS1.S2R34
ROS1	SDC4-ROS1.S4R32.COSF1278
ROS1	SDC4-ROS1.S4R34.COSF1280
ROS1	SLC34A2-ROS1.S13R32.COSF1259
ROS1	SLC34A2-ROS1.S13R34.COSF1261
ROS1	SLC34A2-ROS1.S13R36
ROS1	SLC34A2-ROS1.S4R32.COSF1197
ROS1	SLC34A2-ROS1.S4R34.COSF1198

ROS1	TFG-ROS1.T4R35
ROS1	TPM3-ROS1.T3R36
ROS1	TPM3-ROS1.T7R35.COSF1273
ROS1	ZCCHC8-ROS1.Z2R36

Supplemental Table 3: Response status and concurrent alterations

Arm	Histology	Best response	Best % change	Rearrangement	# cycles	PFS event	PFS Time (mos)	Concurrent Alterations
F	Carcinoma of unknown primary site	SD	+2.4%	EML4-ALK.E6aA20.AB374361 & EML4-ALK.E6bA20.AB374362	1	1	2.3	N/A
F	Mucinous adeno-carcinoma of transverse colon	PR	-61.5%	EML4-ALK.E21A20	6	1	5.3	SMAD4 p.Arg361His<br>SMAD4 p.Arg135Ter<br>APC p.Glu1544Ter
F	Leimyo-sarcoma of abdominal wall arising in mitotically active leimyoma, Müllerian type	CR	-100%	ACTG2-ALK.A2A18	12	0	10.8	N/A

F	Adeno-carcinoma of the rectum	PD	-3.1%	STRN-ALK.S3A20.COSF1430	2	1	1.7	APC p.Arg876Ter<br>TP53 p.Arg282Trp<br>BRAF-BRAF fusion
G	Intrahepatic cholangiocarcinoma	PD	N/A*	GOPC-ROS1.G8R35.COSF1139	1	1	0.5	SMAD4 p.Glu330Lys
G	Adeno-carcinoma of head of pancreas	PD	+5.1%	GOPC-ROS1.G8R35.COSF1139	2	1	1.6	BRCA2 p.Thr3033fs*11<br>BRCA2 pAla938fs*21<br>FBXW7 p.splice site 584+2T>G
G	Low-grade serous adeno-carcinoma of ovary with mucin production	PR	-54.2%	GOPC-ROS1.G4R36.COSF1188	6	0	5.5	NA
G	Adeno-carcinoma of ascending colon	SD	-13%	GOPC-ROS1.G8R35.COSF1139	8	1	6.9	APC p.Leu548fsTer11<br>APC p.Gln1406Ter<br>SMAD4 p.Tyr117fsTer1<br>SMAD4 p.Tyr117Ter<br>TP53 p.Ser94Ter

Supplemental Table 4: Toxicities at least possibly related to treatment

Toxicity Type	Treatment Arm											
	EAY131-F (n=5)				EAY131-G (n=4)				Total (n=9)			
	Grade				Grade				Grade			
	1,2	3	4	5	1,2	3	4	5	1,2	3	4	5
	(n)	(n)	(n)	(n)	(n)	(n)	(n)	(n)	(n)	(n)	(n)	(n)
**Anemia**	1	-	-	-	1	-	-	-	2	-	-	-
**Sinus bradycardia**	2	-	-	-	-	-	-	-	2	-	-	-
**Dry eye**	-	-	-	-	1	-	-	-	1	-	-	-
**Eye disorders - Other, specify**	1	-	-	-	-	-	-	-	1	-	-	-
**Flashing lights**	1	-	-	-	-	-	-	-	1	-	-	-
**Photophobia**	1	-	-	-	-	-	-	-	1	-	-	-
**Abdominal distension**	1	-	-	-	-	-	-	-	1	-	-	-
**Abdominal pain**	1	-	-	-	-	1	-	-	1	1	-	-
**Bloating**	-	-	-	-	1	-	-	-	1	-	-	-
**Constipation**	1	-	-	-	1	-	-	-	2	-	-	-
**Diarrhea**	3	-	-	-	1	-	-	-	4	-	-	-
**Dyspepsia**	-	-	-	-	1	-	-	-	1	-	-	-
**Gastrointestinal disorders - Other, specify**	1	-	-	-	-	-	-	-	1	-	-	-
**Hemorrhoids**	-	-	-	-	1	-	-	-	1	-	-	-
**Nausea**	3	-	-	-	1	-	-	-	4	-	-	-
**Vomiting**	1	-	-	-	1	-	-	-	2	-	-	-
**Fatigue**	2	-	-	-	2	-	-	-	4	-	-	-
**Fever**	-	-	-	-	1	-	-	-	1	-	-	-
**General disorders and administration site conditions - Other, specify**	1	-	-	-	-	-	-	-	1	-	-	-
**Edema limbs**	1	-	-	-	1	-	-	-	2	-	-	-
**Alanine aminotransferase increased**	3	-	1	-	1	-	-	-	4	-	1	-
**Alkaline phosphatase increased**	3	-	-	-	1	-	-	-	4	-	-	-
**Aspartate aminotransferase increased**	2	1	1	-	1	-	-	-	3	1	1	-
**Blood bilirubin increased**	1	-	1	-	-	-	-	-	1	-	1	-

Toxicity Type	Treatment Arm											
	EAY131-F (n=5)				EAY131-G (n=4)				Total (n=9)			
	Grade				Grade				Grade			
	1,2	3	4	5	1,2	3	4	5	1,2	3	4	5
	(n)	(n)	(n)	(n)	(n)	(n)	(n)	(n)	(n)	(n)	(n)	(n)
**Creatinine increased**	-	-	-	-	1	-	-	-	1	-	-	-
**Investigations - Other, specify**	1	-	-	-	-	-	-	-	1	-	-	-
**Lymphocyte count decreased**	2	-	-	-	-	1	-	-	2	1	-	-
**Platelet count decreased**	-	-	-	-	1	-	-	-	1	-	-	-
**Weight gain**	-	-	-	-	1	-	-	-	1	-	-	-
**Weight loss**	-	-	-	-	1	-	-	-	1	-	-	-
**White blood cell decreased**	1	-	-	-	2	-	-	-	3	-	-	-
**Anorexia**	1	-	-	-	1	-	-	-	2	-	-	-
**Hyperglycemia**	1	-	-	-	-	-	-	-	1	-	-	-
**Hyperkalemia**	-	-	-	-	1	-	-	-	1	-	-	-
**Hypoalbuminemia**	2	-	-	-	2	-	-	-	4	-	-	-
**Hypocalcemia**	2	-	-	-	1	-	-	-	3	-	-	-
**Hypoglycemia**	1	-	-	-	-	-	-	-	1	-	-	-
**Hypokalemia**	-	-	-	-	1	-	-	-	1	-	-	-
**Hypomagnesemia**	-	-	-	-	2	-	-	-	2	-	-	-
**Hyponatremia**	-	-	1	-	1	-	-	-	1	-	1	-
**Hypophosphatemia**	-	1	-	-	-	-	-	-	-	1	-	-
**Dysgeusia**	1	-	-	-	-	-	-	-	1	-	-	-
**Headache**	1	-	-	-	1	-	-	-	2	-	-	-
**Acute kidney injury**	-	-	-	-	-	1	-	-	-	1	-	-
**Nasal congestion**	1	-	-	-	-	-	-	-	1	-	-	-
**Rash acneiform**	1	-	-	-	-	-	-	-	1	-	-	-
**Rash maculo-papular**	-	-	-	-	1	-	-	-	1	-	-	-
**Hypertension**	-	-	-	-	1	-	-	-	1	-	-	-
**WORST DEGREE**	3	1	1	-	2	2	-	-	5	3	1	-

Supplemental Table 5:
Frequencies of ALK and ROS1 rearrangements (excluding NSCLC and lymphoma)

	ALK	ROS1
Vendor 1	$1.3x10^{-3}$	$5.6x10^{-4}$
Vendor 2	$7.4x10^{-4}$	$1.6x10^{-4}$
Vendor 3	$6.0x10^{-4}$	$2.0x10^{-4}$
**Combined frequencies from two vendors**		
	$1.0x10^{-3}$	$4.0x10^{-4}$

Supplemental Table 6:
Expanded cohort of *ALK*-rearrangements (excluding NSCLC and lymphoma)

Malignancy	# cases	Proportion of all ALK cases
Thyroid Carcinoma	6	0.162162
Colorectal Adenocarcinoma	5	0.135135
Soft Tissue Tumor	5	0.135135
Pancreatic Adenocarcinoma	4	0.108108
Cancer of Unknown Primary	3	0.081081
Female Genital Tract Malignancy	3	0.081081
Ovarian Surface Epithelial Carcinoma	2	0.054054
Uterine Serous Carcinoma	2	0.054054
Bladder Cancer - Urothelial	1	0.027027
Gastrointestinal Stromal Tumor (GIST)	1	0.027027
Kidney Cancer	1	0.027027
Lung Small Cell Cancer (SCLC)	1	0.027027
Melanoma	1	0.027027
Retroperitoneal or Peritoneal Sarcoma	1	0.027027
Salivary Gland Tumors	1	0.027027

Supplemental Table 7:
Expanded cohort of *ROS1*-rearrangements (excluding NSCLC and lymphoma)

Malignancy	# cases	Proportion of all ROS1 cases
Glioblastoma	8	0.5
Breast Carcinoma	2	0.125
Cancer of Unknown Primary	1	0.0625
Cholangiocarcinoma	1	0.0625
Colorectal Adenocarcinoma	1	0.0625
Pancreatic Adenocarcinoma	1	0.0625
Soft Tissue Tumor	1	0.0625
Thyroid Carcinoma	1	0.0625

Supplemental Table 8: TMB of expanded molecular cohort

TMB	# cases	Proportion
Intermediate	26	0.490566
Low	22	0.415094
High	2	0.037736
QNS	2	0.037736
Indeterminate	1	0.018868

QNS: quantity not sufficient

Supplemental Table 9*:* MSI of expanded molecular cohort

MSI	# cases	Proportion
Stable	49	0.924528
QNS	2	0.037736
High	1	0.018868
Indeterminate	1	0.018868

QNS: quantity not sufficient
