# Supplementary material for: Crizotinib in patients with tumors harboring ALK or ROS1 rearrangements in the NCI-MATCH trial
Source: NPJ Precis Oncol. 2022 Mar 1;6:13. doi: 10.1038/s41698-022-00256-w (PMC8888601; doi:10.1038/s41698-022-00256-w)
Supplement: Supplementary file 3 — REPORTING SUMMARY [file 41698_2022_256_MOESM3_ESM.pdf]

nature portfolio

Corresponding author(s): Dr Aaron Mansfield

Last updated by author(s): 2021 Nov 20

# Reporting Summary

Nature Portfolio wishes to improve the reproducibility of the work that we publish. This form provides structure for consistency and transparency in reporting. For further information on Nature Portfolio policies, see our Editorial Policies and the Editorial Policy Checklist.

## Statistics

For all statistical analyses, confirm that the following items are present in the figure legend, table legend, main text, or Methods section.

| n/a | Confirmed | |
|---|---|---|
| ☐ | ☒ | The exact sample size (*n*) for each experimental group/condition, given as a discrete number and unit of measurement |
| ☐ | ☒ | A statement on whether measurements were taken from distinct samples or whether the same sample was measured repeatedly |
| ☐ | ☒ | The statistical test(s) used AND whether they are one- or two-sided<br>*Only common tests should be described solely by name; describe more complex techniques in the Methods section.* |
| ☐ | ☒ | A description of all covariates tested |
| ☐ | ☒ | A description of any assumptions or corrections, such as tests of normality and adjustment for multiple comparisons |
| ☐ | ☒ | A full description of the statistical parameters including central tendency (e.g. means) or other basic estimates (e.g. regression coefficient) AND variation (e.g. standard deviation) or associated estimates of uncertainty (e.g. confidence intervals) |
| ☐ | ☒ | For null hypothesis testing, the test statistic (e.g. *F*, *t*, *r*) with confidence intervals, effect sizes, degrees of freedom and *P* value noted<br>*Give P values as exact values whenever suitable.* |
| ☒ | ☐ | For Bayesian analysis, information on the choice of priors and Markov chain Monte Carlo settings |
| ☒ | ☐ | For hierarchical and complex designs, identification of the appropriate level for tests and full reporting of outcomes |
| ☒ | ☐ | Estimates of effect sizes (e.g. Cohen's *d*, Pearson's *r*), indicating how they were calculated |

*Our web collection on statistics for biologists contains articles on many of the points above.*

## Software and code

Policy information about availability of computer code

| | |
|---|---|
| Data collection | n/a |
| Data analysis | n/a |

For manuscripts utilizing custom algorithms or software that are central to the research but not yet described in published literature, software must be made available to editors and reviewers. We strongly encourage code deposition in a community repository (e.g. GitHub). See the Nature Portfolio guidelines for submitting code & software for further information.

## Data

Policy information about availability of data

All manuscripts must include a data availability statement. This statement should provide the following information, where applicable:

- Accession codes, unique identifiers, or web links for publicly available datasets
- A description of any restrictions on data availability
- For clinical datasets or third party data, please ensure that the statement adheres to our policy

The data generated or analysed from these clinical trials are included in this published article. The expanded molecular datasets generated or analysed for the current study are available from the corresponding author on reasonable request. The deidentified sequencing data from Caris Life Sciences are owned by Caris Life Sciences. Qualified researchers can apply for access to these summarized data by contacting Joanne Xiu, PhD and signing a data usage agreement. Strata Oncology will provide de-identified molecular results upon request for Strata-referred samples included in this analysis. More specifically, they will provide all prioritized variants for

the ALK/ROS1+ patients, including for the fusions 5'/3' partners, junctions and read support, as well as cancer type, age range, etc. Requests should be addresses to Dan Hovelson, PhD. Tempus has provided summary statistics, including the number of screened patients for this cohort and the count of the specific positives for post-publication replication and verification purposes (Supplemental Table 10). The protocols are available as supplementary materials.

# Field-specific reporting

Please select the one below that is the best fit for your research. If you are not sure, read the appropriate sections before making your selection.

☒ Life sciences ☐ Behavioural & social sciences ☐ Ecological, evolutionary & environmental sciences

For a reference copy of the document with all sections, see nature.com/documents/nr-reporting-summary-flat.pdf

# Life sciences study design

All studies must disclose on these points even when the disclosure is negative.

| | |
|---|---|
| Sample size | Sample size was based on accrual during period that these subarms were open. |
| Data exclusions | One patient enrolled to sub-protocol F was ineligible since treatment was started before the 28 day washout. |
| Replication | Replicates were not included |
| Randomization | Subjects were not randomized |
| Blinding | There was no blinding in this trial |

# Reporting for specific materials, systems and methods

We require information from authors about some types of materials, experimental systems and methods used in many studies. Here, indicate whether each material, system or method listed is relevant to your study. If you are not sure if a list item applies to your research, read the appropriate section before selecting a response.

## Materials & experimental systems

| n/a | Involved in the study | |
|---|---|---|
| ☒ | ☐ | Antibodies |
| ☒ | ☐ | Eukaryotic cell lines |
| ☒ | ☐ | Palaeontology and archaeology |
| ☒ | ☐ | Animals and other organisms |
| ☐ | ☒ | Human research participants |
| ☐ | ☒ | Clinical data |
| ☒ | ☐ | Dual use research of concern |

## Methods

| n/a | Involved in the study | |
|---|---|---|
| ☒ | ☐ | ChIP-seq |
| ☒ | ☐ | Flow cytometry |
| ☒ | ☐ | MRI-based neuroimaging |

## Human research participants

Policy information about studies involving human research participants

| | |
|---|---|
| Population characteristics | Patients with histologically documented solid tumors, lymphomas, or myelomas whose disease had progressed following at least one line of standard systemic therapy or for whom no standard therapy exists were registered on the screening step of the NCI-MATCH protocol to undergo molecular profiling analysis on fresh tumor biopsies. Patients whose tumors were found to harbor actionable ALK or ROS1 rearrangements were offered participation in sub-protocols F or G, respectively. |
| Recruitment | Patients initially had consented to participate in the NCI-MATCH for molecular screening, or were found to have an ALK or ROS1 rearrangement by validated vendors. |
| Ethics oversight | IRB approval was obtained |

Note that full information on the approval of the study protocol must also be provided in the manuscript.

## Clinical data

Policy information about clinical studies

All manuscripts should comply with the ICMJE guidelines for publication of clinical research and a completed CONSORT checklist must be included with all submissions.

| | |
|---|---|
| Clinical trial registration | NCT04439253, NCT04439266 |
| Study protocol | The protocols for the sub-arms are included as supplemental materials |
| Data collection | Individual sites entered relevant clinical data for central review |
| Outcomes | Response rate, progression free survival and overall survival |
